# Virtual Services in the Health Sciences Library: A Handbook

**DOI:** 10.5195/jmla.2022.1640

**Published:** 2022-10-01

**Authors:** Barbara M. Pope

**Affiliations:** 1 bpope@pittstate.edu, Leonard H. Axe Library, Pittsburg State University, Pittsburg, KS.

Health sciences libraries serving universities and medical facilities have long used technology to provide library services, but COVID-19 presented libraries with unique challenges. Libraries shut their doors and adapted to conducting reference, instruction, and outreach, remotely. For some libraries described in *Virtual Services in the Health Sciences Library: A Handbook*, those services had been done in the li-brary; for others, the library had con-sidered adding virtual services. For those libraries that have not made the jump, this text presents strategies to which all health sciences libraries would find a useful reference.

The text presents readers with various perspectives on how medical librarians pivoted existing services to virtual services in the wake of COVID-19. Themes running through the book include relevance and engagement with users, and the challenges of instruction, technology, staff training and scheduling. The book offers librarians perspectives on their response to the shutdown, including innovations in reference services, instruction, and out-reach. Some chapter authors reflect on how the experience allowed them to gather data to inform efforts and improve efficiency and effectiveness during the COVID shutdown and beyond. The text shows that some libraries found the switch to virtual services to be productive and chose to continue services developed during the shutdown.

Various authors, including the following, emphasized their efforts at increasing the library's relevance to users. In chapter 2, Solomon, et al., describe the Countway Library's yearlong brand-building project which occurred prior to COVID-19 (p. 19-20). The brand-building process “define[s] the brand's positioning through an emotional message that reveals what the brand stands for, how it benefits its users, and why they should care” (p. 20). Solomon, et al., adds that brand-positioning helped in communicating information during the COVID-19 shutdown (p. 22-28). However, in chapter 6, Hickner et al., used the COVID-19 shutdown as an opportunity to digitize archival materials and put them online as virtual outreach (p. 75-76). They adapted an annual lecture series to Zoom; because of the online availability, attendance increased 400% (p. 77).

Beyer, et al. describe their library's phased evolution towards virtual reference. Phase one was virtual reference via email; later, they implemented an FAQ (p. 57). In 2013, the library began using video conferencing (p. 57). The library wanted to implement online chat, but could not until staffing made it feasible (p. 61). Online chat launch was planned for Fall 2020, but COVID-19 moved it to March (p. 58). Implementing it “in stages … helped to ensure it was sustainable” (p. 59).

In Chapter 7, Trow relates to readers the challenges of under-funded and under-utilized rural hospital libraries (p. 82). She adds that hospitals “’lack … reserves, … and … must rely on revenues from pricey elective procedures” (p. 81). Trow notes that hospitals' “goal is to … break even, and [ideally] … make money” (p. 82). Hospital libraries do not make money, so are vulnerable to being cut (p. 82). Despite the profit motive, Trow cites the need for “ongoing clinical support for medical decision-making”; she adds that underfunded hospital libraries rely on virtual service, self-service, or clerks and “struggle to demonstrate value and … relevance” (p. 82). In 2019, Trow's library came under scrutiny and changes were needed (p. 83). After surveying users, online resources were acquired to support hospital needs (p. 87-89). The library implemented chat software to track data, thus improving library efficiency and relevance (p. 90-93).

Chapter 4 Harding and Scull looked at textbook affordability. Some pricey health sciences textbooks require costly codes to access content (p. 40). During the COVID-19 shutdown, publishers allowed free access to resources for a limited time, which the authors note was useful, but later, users wanted to “get it back” (p. 41). They add that library purchased ebooks are often unaffordable for libraries (p. 45) “… and that health sciences librarians [must find] … their own solutions” (p. 42). Open educational resources (OER) are viable options to textbooks; despite faculty concerns for the “quality of OER,” studies have shown that students perform well with either option (p. 42-43). They add that “programs [exist] … to incentivize faculty to adopt, adapt, or create OER” (p. 44).

Several authors address challenges such as technology, staff scheduling, virtual reference, and instruction. Scull details how Dartmouth College Biomedical Libraries addressed staffing, particularly information services (reference). In 2019, the libraries finished an information services efficiency and effectiveness improvement project (p. 31-33). Previously, librarians had been “on call” for reference, but other needs had increased, so changes were needed (p. 31-32). Previously, staff used multiple shared email accounts for reference, causing difficulties in collecting data for analysis (p. 32). The staff at Dartmouth Library was trained in LibAnswers and LibChat in 2019 and was able to implement the service during the shutdown (p. 32). All shared email was automatically forwarded to LibAnswers, allowing for capturing data for analysis (p. 32). Reference questions were triaged, and routine questions were answered by information services; patrons needing research assistance were referred to a librarian (p. 32).

Even though library staff had a system in place to “accommodate the change” (p. 35), they scrambled to adapt, which presented technology, supplies, and staff schedule challenges (p. 33). Information services used Springshare as the virtual reference desk (p. 33). The library later assessed virtual reference and found that nearly half of users found “virtual reference to be … convenient” and others “found [it] … productive” (p. 35). Scull added that “[s]taff proved themselves to be highly competent and adaptable …”, but she emphasizes the need to keep in touch with them about “development and training” (p. 37).

Several authors discuss creating or adapting library instruction and issues with library instruction during COVID-19. Evener and Chase (chapter 1) note that their library designed workshops “with the goal of filling [academic skills] gaps students or faculty may have” (p. 6). The “modules are primarily asynchronous,” though some synchronous attendance is required (p. 6). Hickner, et al. adapted instruction to Zoom and coordinated with IT services for technical help (72). They found it helpful to coordinate with others who helped improve their skills “in educational technologies” (p. 73). (Chapter 9) Monnin and Nardella emphasize the differing needs of online and in-person learners (p. 113). They add that “emergency remote teaching” differs from “traditional online … or face-to-face teaching” and that COVID-19 has “unique … challenges, including fewer resources, increased technical difficulties, and less … support” (p. 114).

In the text, *Virtual Services in the Health Sciences Library: A Handbook*, libraries successfully delivered library services and resources by pivoting to virtual services during the COVID-19 shutdown, and as a result improved the efficiency and sustainability of services through innovation, strategy, and planning. Efforts to compare this text to similar titles have yielded a few recent texts, *including Implementing Virtual Ref-erence Services: A LITA Guide* (2013), *Reaching Diverse Audiences with Virtual Reference and Instruction: A Practical Guide for Librarians* (2019), *and Medical Librarian 2.0: Use of Web 2.0 Technologies in Reference Services* (2018). All three titles concern virtual library services, although the first two are not focused on health sciences libraries. Given its practical strategies and engaging text, this reviewer finds *Virtual Services in the Health Sciences Library: A Handbook* to be an inspiring and a highly recommended reference for health sciences libraries.

